# Learning to Localize Cross-Anatomy Landmarks in X-Ray Images with a Universal Model

**DOI:** 10.34133/2022/9765095

**Published:** 2022-06-08

**Authors:** Heqin Zhu, Qingsong Yao, Li Xiao, S. Kevin Zhou

**Affiliations:** ^1^Key Lab of Intelligent Information Processing of Chinese Academy of Sciences (CAS), Institute of Computing Technology, CAS, Beijing 100190, China; ^2^Center for Medical Imaging, Robotics, Analytic Computing & Learning (MIRACLE), School of Biomedical Engineering & Suzhou Institute for Advanced Research, University of Science and Technology of China, Suzhou 215123, China

## Abstract

*Objective and Impact Statement*. In this work, we develop a universal anatomical landmark detection model which learns once from multiple datasets corresponding to different anatomical regions. Compared with the conventional model trained on a single dataset, this universal model not only is more light weighted and easier to train but also improves the accuracy of the anatomical landmark location. *Introduction*. The accurate and automatic localization of anatomical landmarks plays an essential role in medical image analysis. However, recent deep learning-based methods only utilize limited data from a single dataset. It is promising and desirable to build a model learned from different regions which harnesses the power of big data. *Methods*. Our model consists of a local network and a global network, which capture local features and global features, respectively. The local network is a fully convolutional network built up with depth-wise separable convolutions, and the global network uses dilated convolution to enlarge the receptive field to model global dependencies. *Results*. We evaluate our model on four 2D X-ray image datasets totaling 1710 images and 72 landmarks in four anatomical regions. Extensive experimental results show that our model improves the detection accuracy compared to the state-of-the-art methods. *Conclusion*. Our model makes the first attempt to train a single network on multiple datasets for landmark detection. Experimental results qualitatively and quantitatively show that our proposed model performs better than other models trained on multiple datasets and even better than models trained on a single dataset separately.

## 1. Introduction

Accurate and reliable anatomical landmark detection is a fundamental preprocessing step for therapy planning and intervention [1, 2]. It has been proved crucial in many medical clinical scenarios such as knee joint surgery [3], bone age estimation [4], carotid artery bifurcation [5], and pelvic trauma surgery [6]. Furthermore, it plays an important role in medical image analysis [1, 2], e.g., initialization of registration [7, 8] or segmentation [9–11] algorithms.

Manually annotating landmarks by experts is time-consuming and labor intensive; to tackle this challenge, many computer-assisted (CAI) landmark detection methods have been proposed recently. These CAI methods not only automatically localize anatomical landmarks with high accuracy but also save the limited and valuable time of expert radiologists.

Traditional CAI methods are aimed at designing image filters and extracting invariant features, such as SIFT [12]. Liu et al. [13] present a submodular optimization framework to utilize the spatial relationships between landmarks for detecting them. Lindner et al. [14] propose a landmark detection algorithm in the use of supervised random forest regression. However, these methods are less accurate and less robust in comparison to deep neural network methods [15]. Payer et al. [16] propose a novel CNN-based neural network which integrates spatial configuration into the heat map and demonstrates that, for landmark detection, local features are accurate but potentially ambiguous, while global features eliminate ambiguities but are less accurate [17, 18]. Yang et al. [19] propose a deep image-to-image network built up with an encoder-decoder architecture for initializing vertebra locations, which are evolved with another ConvLSTM model and refined by a shape-based network. Recently, Lian et al. [20] develop a multitask dynamic transformer network for bone segmentation and large-scale landmark localization with dental CBCT, which also makes use of global features when detecting landmarks.

It is a challenging task to detect landmarks accurately and robustly. Ambiguity always occurs when detecting landmarks in locally similar structures, which makes it hard to locate accurate and less-ambiguity landmarks [16]. To deal with this, the global context information should be taken into consideration and integrated with local features for robust landmark detection. A great number of methods empower the globallocal architecture and make great success. Chen et al. [21] propose a 3D local semantic network combined with a 2D long-range contextual network for 3D vertebra localization and achieve state-of-the-art performances. Payer et al. [16] split the localization task into two simpler subproblems: the first step is dedicated to locally accurate candidate predictions and the second step is to eliminate ambiguities by using the spatial context information. Lian et al. [20] concurrently segment craniomaxillofacial (CMF) bones and localize large-scale landmarks by using a transformer [22] which has an extraordinary power of modeling long dependencies. In addition, medical images have various anatomical regions such as the head, hand, chest, and pelvis. The existing method is highly specialized for a single domain associated with a particular anatomical region. Despite that SCN [16] is capable of detecting landmarks in head, hand, and chest datasets, it needs to be trained, which costs more time and storage. Therefore, developing a universal model that detects crossanatomy landmarks is promising and desirable [23–25].

In this work, we develop a powerful model for detecting the landmarks associated with different anatomies (head, hand, chest, and pelvis), each exemplified by a dataset, which overcomes the abovementioned limitations of the existing methods and demonstrates state-of-the-art detection accuracy. Our approach benefits from “Big Data,” implicitly models the relevance among different anatomical regions, learns once on multiple datasets, and works for all domains, that is “You Only Learn Once.” To explore the common knowledge among the seemingly different datasets, our model utilizes the aggregation of all input images from different datasets at the same time to train domain-specific parameters and domain-shared parameters. To the best of our knowledge, this marks the *first such attempt* for anatomical landmark detection.

**Figure 1:**
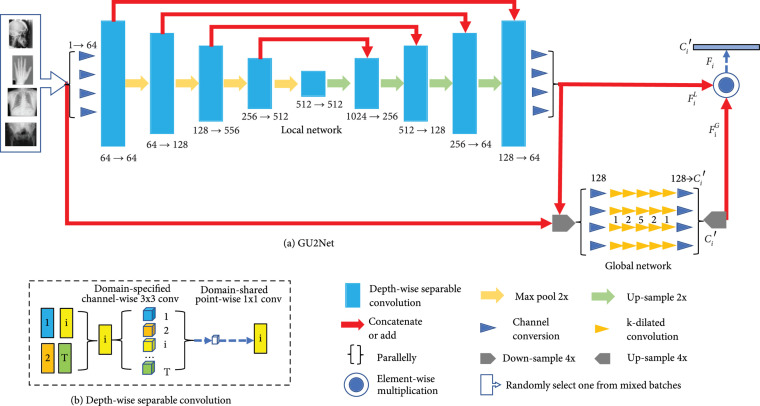
(a) The structure of our GU2Net model consists of two parts, namely, the local network and the global network. The local network is a U-Net structure with each convolution being replaced with depth-wise separable convolution which consists of channel-wise convolution and point-wise convolution. The global network is a parallel-duplicated sequential of five dilated small-kernel-size convolutions. (b) Depth-wise separable convolution. Each 3×3 convolution is followed by batch normalization and leaky ReLU. The global network takes the four-time downsampled image and local heat map as input and outputs the four-time upsampled heat map.

Our model, named as global universal U-Net (GU2Net), consists of two parts: a local network and a global network (see Figure 1). The local network is inspired by the universal design of Huang et al. [23]. We replace each standard convolution in U-Net with a *depth-wise separable convolution* [26, 27]. The depth-wise separable convolution consists of *channel-wise convolution* and *point-wise convolution*, which model domain-specific and domain-shared parameters, respectively, and have fewer parameters than a standard convolution [23]. Then, we duplicate the channel-wise convolution in parallel for every input dataset. The local network extracts local features, which are mostly accurate but still possibly ambiguous for landmarks. We follow the globallocal schema [16, 20, 21, 28] and introduce the global network to further integrate global context information and guide the local network to detect more accurate and less ambiguous landmarks.

In sum, we make the following contributions and our model is publicly available at https://github.com/MIRACLE-Center/YOLO_Universal_Anatomical_Landmark_Detection:

(i) The *first attempt* in the literature, to the best of our knowledge, to develop a multidomain landmark detection model that works for multiple datasets and across different anatomical regions, unleashing the potential of “bigger data”
(ii) *State-of-the-art performances* of detecting a total of 72 landmarks based on four X-ray datasets of the head, hand, chest, and pelvis, totaling 1710 images but using only one model which needs fewer parameters
(iii) *A novel landmark detection method* that integrates local features with global context information to detect more accurate and precise landmarks

This work is based on a preliminary work [29] published as a conference paper (the reviewers’ comments and the authors’ feedback can be found in https://miccai2021.org/openaccess/paperlinks/2021/09/01/531-Paper0185.html.). In this paper, the following improvements have been proposed for extending a previous work:

(i) In [29], we proposed a novel universal model and evaluated the model on three different anatomical regions (i.e., head, hand, and chest). Here, to demonstrate the effectiveness and simplicity of extending to new domains of our model, we introduce an additional pelvis dataset for evaluation
(ii) In this paper, we investigate the benefit of common knowledge learning through domain-shared parameters by evaluating the performances of our model trained on different numbers of domains
(iii) We also investigate the domain knowledge learning through domain-specific parameters by exchanging the domain-specific parameters between two domains

## 2. Results

### 2.1. Framework

The framework of our universal landmark detection method is a full CNN-based network (see Section 4.3), which takes mixed batches of different datasets as inputs and generates heatmaps to locate the landmarks. It consists of a global network and a local network, which integrates global context information to local features for more accurate localization.

In our experiments, we utilize four X-ray datasets (head, hand, chest, and pelvis) with various anatomical regions detailed in Section 4.1. We carry out qualitative and quantitative experiments on these four datasets to compare our proposed method with the state-of-the-art methods from different perspectives in two metrics: mean radial error (MRE) and successful detection rates (SDR). MRE is defined as $\text{MRE}=\left(1/N\right)\sum_{i=1}^{N}{\left\|x_{i}-{\tilde{x}}_{i}\right\|}_{2}$, where $x_{i}$ is the coordinates of ground truth landmark and ${\tilde{x}}_{i}$ is the coordinates of the predicted landmark. SDR within k (mm or px) is defined as ${\text{SDR}}_{k}=\left(1/N\right)\left|\left\{\left.i\right|{\left\|x_{i}-{\tilde{x}}_{i}\right\|}_{2}<k,i=1,\cdots ,N\right\}\right|$. Except as otherwise noted, models are learned only once on all datasets which are randomly mixed per batch. Evaluation is carried out on a single dataset separably for each method. Furthermore, we conduct detailed parameter analysis to figure out how our GU2Net learns common knowledge and benefits from universal learning. Unless otherwise specified, all experiments share the same setting described in Section 4.5.

### 2.2. Performance Analysis

The evaluation results of our model’s performance and detection accuracy additionally compared with other methods are shown in Table 1. We compare our GU2Net with methods that are designed and trained for a specific domain on a single dataset, such as random forest regression voting proposed by Lindner et al. [14] and spatial configuration network introduced by Payer et al. [16]. Moreover, a U-Net trained on the mixed four datasets is demonstrated as the baseline for multidomain landmark detection. The global network takes a downsampled image and local heat map as input, fusing features in two ways: concatenation and addition. The two versions of GU2Net are denoted as GU2Net-cat and GU2Net-add, respectively.

**Table 1:** Detection accuracy comparison with MRE and SDR metrics of different models on head, hand, chest, and pelvis datasets.

Models	Head					Hand				
	MRE	STD	SDR (%)			MRE	STD	SDR (%)		
	mm	mm	2 mm	3 mm	4 mm	mm	mm	2 mm	4 mm	10 mm
Ibragimov et al. [30]∗	1.84	—	68.13	79.77	86.87	—	—	—	—	—
Štern et al. [31]${}^{*}$	—	—	—	—	—	0.80	0.91	92.20	98.45	99.83
Lindner et al. [14]${}^{*}$	1.67	—	70.65	82.17	89.85	0.85	1.01	93.68	98.95	99.94
Urschler et al. [32]${}^{*}$	—	—	70.21	82.08	89.01	0.80	0.93	92.19	98.46	99.95
Payer et al. [16]${}^{*}$	—	—	73.33	83.24	89.75	*0.66*	*0.74*	94.99	99.27	*99.99*
U-Net [33]^†^	3.03	8.71	64.93	80.29	88.27	4.86	18.60	78.35	90.43	93.74
GU2Net-cat^†^	*1.51*	*2.41*	78.67	89.77	94.67	0.85	3.01	95.20	99.22	99.76
GU2Net-add^†^	*1.51*	2.46	*78.74*	*90.26*	*94.78*	0.81	2.21	*95.23*	*99.38*	99.79
Models	Chest					Pelvis				
	MRE	STD	SDR (%)			MRE	STD	SDR (%)		
	px	px	3 px	6 px	9 px	px	px	3 px	6 px	9 px
U-Net [33]^†^	63.61	119.93	24.24	52.53	65.66	5.82	16.71	54.82	82.41	89.52
GU2Net-cat^†^	*4.59*	*8.36*	*53.28*	77.53	89.65	*5.68*	*15.27*	56.84	*83.68*	*90.00*
GU2Net-add^†^	4.74	9.24	52.34	*80.73*	*89.84*	8.79	25.72	*59.44*	83.06	89.44

${}^{*}$The performances that are copied from the original paper; the model is learned on a single dataset separately. ^†^The model is learned on the mixed four datasets. —: no experimental results can be found in the original paper. In each column, the best results are in italics and the second-best results are underlined.

As Table 1 shows, on the head dataset, our method achieves the best accuracy with all thresholds (2 mm, 3 mm, and 4 mm) and obtains an MRE of 1.51±2.41 mm, behaving much better than U-Net, which is also learned on the mixed multiple datasets. It even beats the other state-of-the-art methods marked with ∗ that are learned on a single dataset for a specific domain. Within 2 mm, our model achieves the best SDR of 78.67%, outperforming the previous state-of-the-art method [16] by 5.34%. Such an improvement is consistent among SDRs at other distance thresholds. On the hand dataset, our method reaches the best accuracy of 95.23% within 2 mm, which is far ahead of other models learned on the single-hand dataset. When comparing our GU2Net and U-Net which are trained on mixed multiple datasets for multidomain learning, our method performs better than U-Net in all metrics by a huge margin. Our method obtains an MRE of 4.59±8.36 px on the chest dataset and 5.68±15.27 on the pelvis dataset. In summary, our proposed method generally outperforms the state-of-the-art methods learned on a single dataset. Compared to U-Net learned on multiple datasets, our model outperforms U-Net on each metric by a huge gap, especially under high-precision conditions, which is evident from the SDR values within say 2 mm, 4 mm, 3 px, and 6 px. U-Net behaves extremely worse on the chest dataset as the model even does not converge and obtains an MRE of 63.61 mm, showing that UNet has a limited power in multidomain learning while our model is capable of learning on multiple datasets and achieves admirable performances. When comparing the feature fusion method concatenation to addition, GU2Net-cat performs a little better on chest and pelvis datasets, but worse on head and hand datasets, which indicates that the performances are mainly determined by the architecture of our GU2Net, not the feature fusion method.

### 2.3. Ablation Study

In order to demonstrate the effectiveness of our local network $\varphi_{LN}$ and global network $\varphi_{GN}$, we perform an ablation study on the mixed dataset by merging the four datasets together. There are total 250+300+75+36=661 images for testing. The average MRE and SDR on the mixed dataset are adopted as metrics. We evaluate the performance on U-Net, $\varphi_{LN}$ (with local network only) and $\varphi_{GN}$ (with global network only), and the proposed GU2Net-cat.

**Figure 2:**
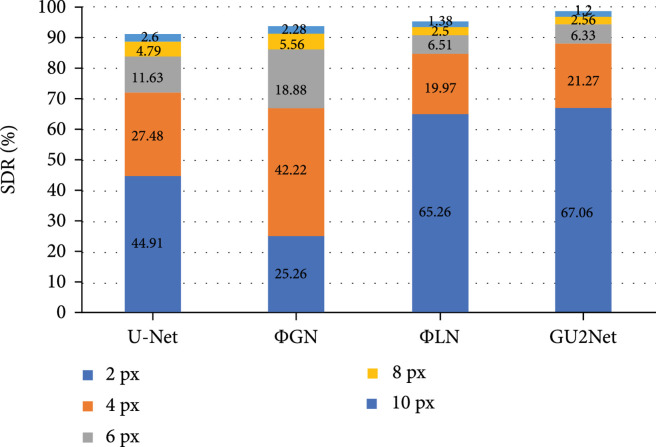
Ablation study of our proposed model with local network and global network.

As shown in Figure 2, when comparing $\varphi_{LN}$ with U-Net, it is evident that depth-wise separable convolution in the local network improves the model’s performance by a large margin. Thus, the architecture of the local network is more capable of learning across anatomies. By comparing GU2Net with $\varphi_{LN}$ and $\varphi_{GN}$, we observe much improvement of SDR within 4 px, which demonstrates the effectiveness of fusing local information and global information. Thus, global information and local information are equally important for accurate localization of anatomical landmarks. Since U-Net only has domain-shared parameters and $\varphi_{GN}$ only contains domain-specific parameters, their performances are the worst and fall behind $\varphi_{LN}$ while GU2Net have both types of parameters, by a huge gap. It is worth mentioning that the parameter number of our GU2Net is around 5 M, one-third of that of U-Net, which demonstrates the superiority of our architecture and the indispensability of both shared and domain-specific parameters.

**Figure 3:**
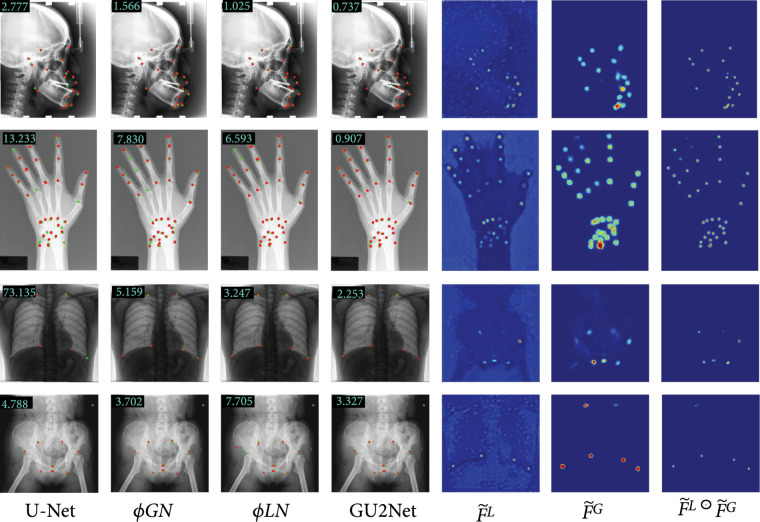
Selected visual examples of predicted landmarks and generated feature heat map on head, hand, chest, and pelvis datasets. The first four columns are landmarks predicted by four different models, where the red points (red circle) are the learned landmarks and the green points (green circle) are the ground truth labels, with the MRE value being displayed on the top-left corner of the image for reference. The last three columns are local heat map ${\tilde{F}}^{L}$, global heat map ${\tilde{F}}^{G}$, and final heat map $\tilde{F}$ generated by our model.

To qualitatively show the superiority of our GU2Net, we further visualize the predicted landmarks and generated feature heat maps in Figure 3. The MRE value is displayed on the top-left of the image for reference. It is clear that our model’s results have more overlap regions of red and green points than other models, which also can be verified according to the MRE values. Per the last three columns, the local network generates accurate landmarks while the global network generates rough landmarks. The final accurate and unambiguous heat map is obtained by multiplying the two heatmaps.

### 2.4. Domain-Shared Parameter Analysis of Common Knowledge Learning

**Figure 4:**
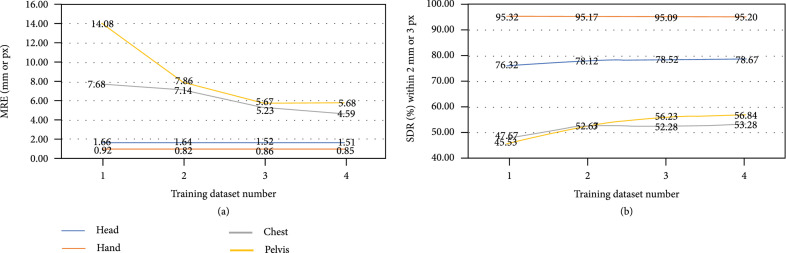
Visualization of performances of our proposed GU2Net trained on different numbers of datasets. (a) Shows the MRE results while (b) shows the SDR within 2 mm or 3 px.

In order to demonstrate the effectiveness and necessity of learning on mixed multiple datasets, we perform domain-wise experiments by changing the number of mixed datasets, which explains the effect of domain-shared point-wise convolution for common knowledge learning. Specifically, we train our proposed neural network on the (1) single dataset, (2) different combinations of two datasets, (3) different combinations of three datasets, and (4) four datasets and test it on the single dataset. Observing that (2) and (3) have different combinations of datasets and thus different experimental results, we display the average results for one specific dataset as follows. For instance, when training the model on two mixed datasets, the test results of the head dataset have three values for each metric: (head, hand), (head, chest), and (head, pelvis), which is the same with other datasets. When training the model on three mixed datasets, we also take the average of three result values as the final result, namely, (head, hand, and chest), (head, hand, and pelvis), and (head, chest, and pelvis) for the head dataset. As shown in Table 2, GU2Net trained on 4 datasets (all datasets) obtains the best performances on head and chest datasets in all metrics and a little worse than the best on hand and pelvis datasets under measurement of MRE. GU2Net trained on 4 datasets for multidomain beats GU2Net trained on a single dataset for a specific domain by a huge gap in all metrics except MRE on the hand dataset (this is possibly due to the enough amount of hand images for training a specialized network), such as 4.59 px of MRE on the chest dataset, 78.67% of SDR within 2 mm on the head dataset, which demonstrates training on multiple datasets boosts our model to learn common knowledge among different datasets and further improve performances on all datasets. Moreover, we visualize the results in Figure 4 to have an intuitive insight. With the number of trained datasets increasing from 1 to 4, MRE and SDR metrics generally become better on each dataset.

**Table 2:** Evaluation of our proposed GU2Net that is trained on different numbers of mixed datasets.

Dataset number	Head		Hand		Chest		Pelvis	
	MRE	SDR (%)	MRE	SDR (%)	MRE	SDR (%)	MRE	SDR (%)
	mm	2 mm	mm	2 mm	px	3 px	px	3 px
1	1.66	76.32	0.92	*95.32*	7.68	47.67	14.08	45.53
$2^{*}$	1.64	78.12	*0.82*	95.17	7.14	52.67	7.86	52.63
$3^{*}$	1.52	78.52	0.86	95.09	5.23	52.28	*5.67*	56.23
4	*1.51*	*78.67*	0.85	95.20	*4.59*	*53.28*	5.68	*56.84*

${}^{*}$Values are averaged of three experimental results (detailed in Section 2.3. The original results before averaging are available in supplementary materials in Table S1). In each column, the best results are in italic and the second-best results are underlined.

### 2.5. Domain-Specific Parameter Analysis of Domain Knowledge Learning

Our universal model not only learns common knowledge through domain-shared parameters but also learns domain-specific knowledge for each anatomy. To demonstrate this, we carry out cross-anatomy tests and visualize the final feature maps after using t-SNE to reduce dimensionality.

**Figure 5:**
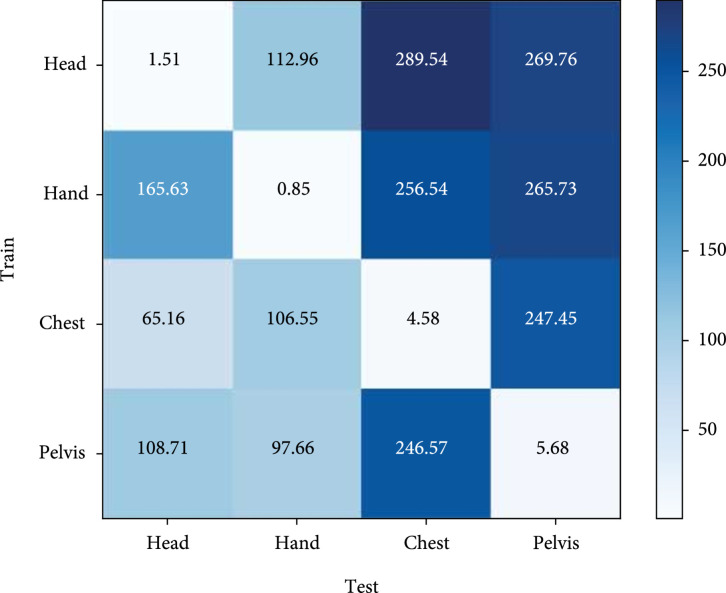
Visualization of the cross-anatomy test of our proposed GU2Net trained on mixed datasets under the measurement of MRE (mm or px).

We firstly train GU2Net on four mixed datasets of different anatomies, which results in domain-shared parameters for all anatomies and four kinds of domain-specific parameters for each anatomy. Then, we test each anatomy by using domain-shared parameters and one of the four kinds of domain-specific parameters. The MRE (mm or px) results are illustrated in Figure 5. As it shows, when training domain-specific parameters on $T_{i}\in \left\{\text{head},\text{hand},\text{chest},\text{pelvis}\right\}$ the anatomy and testing on the $T_{i}$ anatomy, the MRE results are relatively low and correct, namely, 1.51 mm for the head, 0.85 mm for the hand, 4.59 px for the chest, and 5.68 px for the pelvis. However, when training domain-specific parameters on the $T_{i}$ anatomy and testing on the $T_{j}\left(T_{j}\neq T_{i}\right)$ anatomy, the MRE results are extremely high. For instance, when trained on the head and tested on the hand, the MRE is 112.96 mm, which indicates the wrong inference results. From what has been discussed, we can safely draw a conclusion that our universal model learns domain-specific knowledge through domain-specific parameters, which are only applied for the corresponding anatomy and cannot be shared with other anatomies.

**Figure 6:**
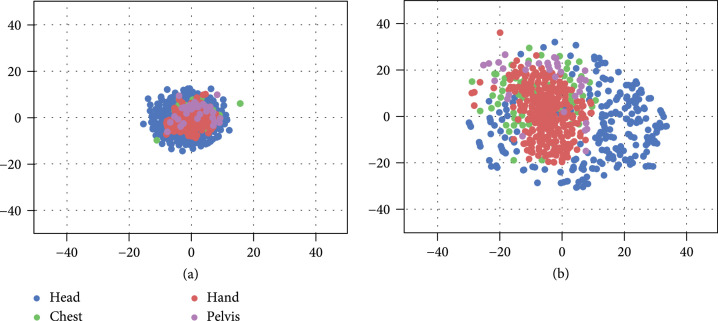
Visualization of the absolute difference of output feature maps and ground truth Gaussian maps. The results are processed by t-SNE for dimensionality reduction. (a) Shows the results of GU2Net while (b) shows the results of U-Net, which are both trained on mixed datasets.

We further visualize the absolute difference of output feature maps, which are from GU2Net and U-Net, and ground truth Gaussian maps using t-SNE. As Figure 6 shows, points with different colors represent different anatomies. All kinds of points of GU2Net are more tightly clustered around (0,0) than those of U-Net, which demonstrates that GU2Net utilizes domain-specific parameters to generate mainly accurate and same feature maps as ground truth Gaussian maps for each anatomy.

## 3. Discussion

The goal of this research is to establish a universal model that is trained on multiple datasets once and is capable of inferring images from these datasets for anatomical landmark detection. To achieve this goal, GU2Net is designed with two key components: depth separable convolution and global-local architecture. Depth separable convolution empowers the local network to learn domain-specific knowledge and domain-shared knowledge for multiple anatomies, which also costs fewer parameters and storage compared to standard convolution. The global-local architecture jointly makes use of global context information and local features to detect accurate and less ambiguous landmarks, which improves the performance compared to a single local network. Benefiting from the elaborated design of architecture, GU2Net can be easily extended to more diverse anatomies, which is evident from the experiments of different training datasets in Table 2. For example, we can finetune a trained GU2Net by freezing domain-shared parameters and adding domain-specific parameters for an unseen anatomical region. Common knowledge does exist among different anatomies and helps GU2Net behave better than models trained on a single dataset for a specific domain.

While our universal model is demonstrated significantly better compared to the existing method, it still has several limitations. Our model is unable to detect landmarks when the input image comes from an unknown anatomy because there are no corresponding domain-specific parameters in our model to react properly. Therefore, our model is perfectly used for situations that include several predetermined anatomies to detect more accurate landmarks. However, it is possible to extend new anatomy by adding the new dataset to the mixed training datasets. Another limitation is that it is hard to unify 2D data and 3D data and achieve better performances at the same time. To unify 2D data and 3D data, domain-specific layers (convolution, batch norm, and max pool) should match the related data while domain-shared layers (only 1×1 point-wise convolution) are converted to 3D point-wise convolutions with no side effects. When passing to 3D point-wise convolution, a 2D slice is viewed as a one-slice 3D volume. However, with a huge gap of slice number between 2D data and 3D data, the unified model learns little common knowledge and results in poor performances. Future work may extend to unify different modalities. Despite the abovementioned limitations, our model unifies diverse anatomies and archives state-of-the-art performances by utilizing common knowledge. We believe that this work evokes the exploration of common knowledge learning for a unified model.

## 4. Materials and Methods

### 4.1. Datasets

#### 4.1.1. X-Ray Cephalogram Dataset

The cephalogram dataset is an open-source dataset firstly released in the ISBI 2015 Cephalometric X-ray Image Analysis Challenge [34]. There are 400 2D X-ray cephalogram images in this dataset, with 150 images for training and the remaining 250 images for testing. Each image is of size 1935×2400 with a physical resolution of 0.1 mm×0.1 mm. We resize the original image to the size of 416×512 for computational efficacy during training and testing. Each image has 19 landmarks that are manually labeled by a senior expert and a junior expert. We use the averaged coordinates to form the final landmark labels according to Payer et al. [16].

#### 4.1.2. X-Ray Hand Dataset

The hand dataset is also a publicly available dataset of radiographs maintained by the Digital Hand Atlas Database System (https://ipilab. usc.edu/research/baaweb/). This dataset contains 895 2D X-ray images with various image sizes. Therefore, we resize each image to a fixed size of 352×512. We split the whole dataset into two parts which resulted in 595 training images and 300 testing images. Since the source files are in jpg format and provide no information about physical resolution, we normalize the image resolution according to wrist widths as suggested by [14]. More specifically, assuming that the width of the wrist is 50 mm, with the two endpoints of the wrist being a,b and the pixel distance being $d_{\text{pixel}}$, the physical distance $d_{\text{physical}}$ can be formulated as

(1)dphysical=50a−b2⋅dpixel.

We use a total of 37 landmarks manually labeled by Payer et al. [16] and the coordinates of a,b can be directly obtained from the first and the fifth points of the 37 landmarks.

#### 4.1.3. X-Ray Chest Dataset

Our chest dataset is a subset of Pulmonary Chest X-Ray Abnormalities (https://www.kaggle.com/nikhilpandey360/ chest-xray-masks-and-labels). We select the China subset and exclude the cases labeled as abnormal lungs (which means diseased lungs in the original dataset) to form our experimental dataset, resulting in a total of 279 images. The first 204 images are used for training and the remaining 75 images are used for testing. We manually label 6 landmarks in each image. The left three landmarks lie on the top, the bottom, and the right boundary of the right lung. Correspondingly, the right three landmarks lie on the top, the bottom, and the left boundary of the left lung. The 6 landmarks appropriately determine the shape of the two lungs (see Figure 3). We also resize the input image to a fixed size of 512×512. Since all images are in a png format and the physical resolution is not known, we use a pixel distance to measure performances in this dataset.

#### 4.1.4. X-Ray Pelvis Dataset

We use an in-house pelvis dataset of 136 X-ray images with 10 manually annotated landmarks. Same as the chest dataset, the images in the pelvis dataset have various shapes and no information about physical resolution. Therefore, we resize the images to a fixed shape of 512×512 and use a pixel distance for performance evaluation. We partitioned this dataset which resulted in 100 training images and 36 testing images.

### 4.2. Problem Definition

The goal is to develop one landmark detection model trained on mixed t datasets $\left\{T_{1},T_{2},\cdots ,T_{t}\right\}$, which are potentially from various anatomical regions in different dimensions. For an input image $X_{i}\in R^{C_{i}\times W_{i}\times H_{i}}$ and corresponding landmark coordinates $\left\{x_{ik}\right\}\left(k\in \left[1,2,\cdots ,{\text{C}}_{i}^{\prime }\right]\right)$ from dataset $T_{i}$, the heat map image is a concatenation of the single landmark’s heat map. The *k*th landmark’s heat map $Y_{ik}\in R^{C_{i}^{\prime }\times W_{i}\times H_{i}}$ is formulated as the Gaussian function:

(2)Yikx=γ2πσexp−x−xik22σ2,

where $C_{i}$ is the number of channels on the input image from dataset $T_{i}$ (i.e., $C_{i}=1$ for an X-ray image); $C_{i}^{\prime }$ is the number of channels on the output heat map, namely, the number of landmarks; $D_{i}$, $W_{i}$, and $H_{i}$ are the depth, width, and height of image $X_{i}$, respectively. γ is a scaling factor introduced to avoid numerical instabilities as suggested by [16], which ensures that the heat map has a [0,1] value range. σ is a fixed hyperparameter.

### 4.3. Global Universal U-Net

Aiming at detecting accurate landmarks on multiple domains, our proposed GU2Net architecture is designed as a combination of a local network and a global network. As shown in Figure 1, the local network is a U-like convolution network composed of depth-wise separable convolutions, while the global network is composed of parallel-duplicated dilated convolutions.

#### 4.3.1. The Local Network

The local network $\varphi_{LN}$ (see Figure 1(a)) is a convolutional network that extracts local features and generates a local heat map, which is used to determine the accurate location of landmarks. Each convolution block is a depth-wise separable convolution that consists of domain-specific channel-wise convolution and domain-shared point-wise convolution, followed by batch normalization and leaky ReLU. Each dataset is assigned a different channel-wise convolution separately, in which parallel extracts domain-specific features from the input feature map and feeds the extracted features into point-wise convolution shared by all datasets to integrate local features from different datasets and learn common knowledge for better performance. The local heat map ${\tilde{F}}_{i}^{L}$ is generated as the following formula:

(3)F~iL=ϕLNXi;θdiL,θsL,

where $\theta_{di}^{L}$ is the parameter of domain-specific channel-wise convolution corresponding to dataset $T_{i}$ and $\theta_{s}^{L}$ is the parameter of domain-shared point-wise convolution served for all datasets. Such structure allows a perfect match for the multidomain landmark detection; moreover, there is an extreme decrease of the parameter number and computation burden compared with t parallel-duplicated standard convolutions. In depth-wise separable convolution, considering an N-channel input feature map and an M-channel output feature map, we firstly apply N*-*channel-wise filters in the shape of $R^{3\times 3}$ to each channel and concatenate the N-output feature maps. Secondly, we apply M-point-wise filters in shape of $R^{1\times 1\times N}$ to output the feature maps of M-channels [23]. Accordingly, the total number of parameters is 9×t×N+N×M, while it is 9×t×N×M for t standard 3×3 convolutions.

#### 4.3.2. The Global Network

Global structural information provides an insight of the rough location of landmarks [16, 20, 21, 28] for accurate landmark detection, which motivates us to design an additional global network $\varphi_{GN}$. $\varphi_{GN}$ is composed of a sequence of dilated convolutions. Each dilated convolution has a 3×3 kernel size and followed by batch normalization and ReLU. Since the global context from different datasets varies a lot in appearance, we duplicate the dilated convolutions for each dataset (see Figure 1), resulting in domain-specific parameters ${\theta_{di}}^{G}.\varphi_{GN}$ which takes image $X_{i}$ and local feature ${{\tilde{F}}_{i}}^{L}$ as input and aggregates the global information at a coarse-grained scale, flowing global heat map ${{\tilde{F}}_{i}}^{G}$:

(4)F~iG=ϕGNXi,F~iL;θdiG.

### 4.4. Loss Function

As illustrated in Figure 1(a), we multiply the local heat map ${{\tilde{F}}_{i}}^{L}$ and global heat map ${{\tilde{F}}_{i}}^{G}$ element wise, resulting in final heat map ${\tilde{F}}_{i}={{\tilde{F}}_{i}}^{G}⊙{{\tilde{F}}_{i}}^{L}$, where ⊙ is the element-wise multiplication. In the training stage, we penalize the final heat map ${\tilde{F}}_{i}$ and the ground truth $Y_{i}$ (defined in equation (2)):

(5)Li=∑y∈Yi,f∈F~i−y log f−1−ylog1−f.

In the inference stage, the kth landmark is obtained after finding the maximum location of the kth channel in final heat maps ${\tilde{F}}_{i}$.

(6)landmarkk=argmaxF~ik.

### 4.5. Implementation and Setting

All neural networks are implemented in PyTorch 1.3.0 and run on a TITAN RTX GPU with the CUDA version being 11. The kernel sizes of convolution are 3×3 and 1×1 with 3×3 convolution followed by batch normalization [35] and leaky ReLU in the local network and ReLU in the global network [36]. There are 5 dilated convolutions for each dataset in the global network, with dilations being [1, 2, 5, 2, 1]. Due to the lower accuracy and harder training of directly regressing landmark coordinates, we instead predict a Gaussian heat map, which retains the probability distribution of the landmark in each pixel with σ set to 3.

We perform on-the-fly data augmentation when loading image data. The intensity value is normalized by the Z-score normalization. The input images are randomly rotated by 2 degrees with 0.1 probability and translated by 10 pixels in each direction with 0.1 probability. When training networks, we set the batch size to 4 and learning rate to $\left[1e-4,1e-2\right]$. The binary crossentropy (BCE) loss and an Adam optimizer are used to train the network up to 100 epochs, and a cyclic scheduler [37] is used to decrease the learning rate from 1e−2 to 1e−4 dynamically. The inference model is chosen as the one with minimum validation loss for evaluation.

## Data Availability

All data are available within the article and supplementary files or available from the authors upon request.

## Supplementary Materials

Supplementary Materials Table S1: original results for domain-shared parameter analysis.
