# Supplementary material for: Learning to Localize Cross-Anatomy Landmarks in X-Ray Images with a Universal Model
Source: BME Front. 2022 Jun 8;2022:9765095. doi: 10.34133/2022/9765095 (PMC10521670; doi:10.34133/2022/9765095)
Supplement: Supplementary Materials — Table S1: original results for domain-shared parameter analysis. [file 9765095.f1.pdf]

Table S1: Original results for domain-shared parameters analysis.

Datasets	Head		Hand		Chest		Pelvis	
	MRE	SDR(%)	MRE	SDR(%)	MRE	SDR(%)	MRE	SDR(%)
	mm	2mm	mm	2mm	px	3px	px	3px
Head, Hand	1.66	76.32	0.83	95.41	-	-	-	-
Head, Chest	1.60	77.31	-	-	10.37	55.67	-	-
Head, Pelvis	1.72	77.98	-	-	-	-	9.13	50.53
Hand, Chest	-	-	0.84	94.98	5.23	52.33	-	-
Hand, Pelvis	-	-	0.78	95.11	-	-	6.85	57.37
Hand, Pelvis	-	-	-	-	5.85	50.00	6.85	57.37
Head, Hand, Chest	1.54	77.79	0.84	95.40	5.57	57.33	-	-
Head, Hand, Pelvis	1.45	80.13	0.82	95.23	-	-	5.20	62.63
Head, Chest, Pelvis	1.56	77.64	-	-	5.54	50.51	6.61	50.26
Hand, Chest, Pelvis	-	-	0.92	94.65	4.57	48.99	5.21	55.79
